# Separating People from Pollution: Individual and Community Interventions to Mitigate Health Effects of Air Pollutants

**DOI:** 10.1289/ehp.119-a34a

**Published:** 2011-01

**Authors:** Cynthia Washam

**Affiliations:** **Cynthia Washam** writes for *EHP, Oncology Times*, and other science and medical publications from South Florida

Efforts to minimize people’s exposure to air pollution historically have focused on curbing emissions from tailpipes and smokestacks. But increases in vehicle-kilometers traveled—that is, more cars spending more time on the road—have tempered that effect. Moreover, residential areas, hospitals, and schools often are built adjacent to main traffic arteries, where emissions are highest. An international group of public health researchers now says it’s time to start separating people from sources of air pollution as a means of protecting public health **[*EHP*** **119(1):29–36; Giles et al.]**.

Air pollution can cause myriad cardiovascular and respiratory problems including asthma, bronchitis, and heart disease. Outdoor air pollutants can easily migrate indoors, and most exposure to ambient air pollution occurs inside buildings. Recent research indicates that people living near congested highways face a greater risk of such diseases and that moving to a less-polluted neighborhood lowers their risk.

The authors describe “promising and largely unexplored” approaches to reducing the health impact of air pollution through interventions targeted at communities and at individuals. They base their recommendations on published studies and discussions from a 2009 workshop on this topic held in Vancouver, Canada.

The authors argue that cities can improve residents’ health by considering air quality during land-use planning. For example, creating high-density, mixed-use areas would enable more people to walk or bicycle to work, school, and shops, thereby reducing emissions and encouraging more exercise; ideally, safe pedestrian and cycling greenways would be located away from traffic. For longer-distance travel, the authors suggest low-emission public transit. And in areas where wood burning is an important heating method, woodstove exchange programs can help residents acquire cleaner-burning stoves affordably.

Risk factors for heart disease include a sedentary lifestyle, obesity, and a high-sodium diet. Therefore, the authors posit that another approach to reducing a person’s risk of being affected by air pollution is to minimize one’s overall risk of heart disease. This could involve interventions that encourage people to eat a diet rich in omega-3 fatty acids and antioxidants and to get regular exercise. However, because pollution levels vary even within cities, exercise should be planned to minimize exposure. Variations occur by season, with ozone being higher in the summer and particulates from woodstoves higher in the winter, for example. Traffic-related pollutants also spike during rush hour and are higher in heavily traveled areas.

## Figures and Tables

**Figure:**
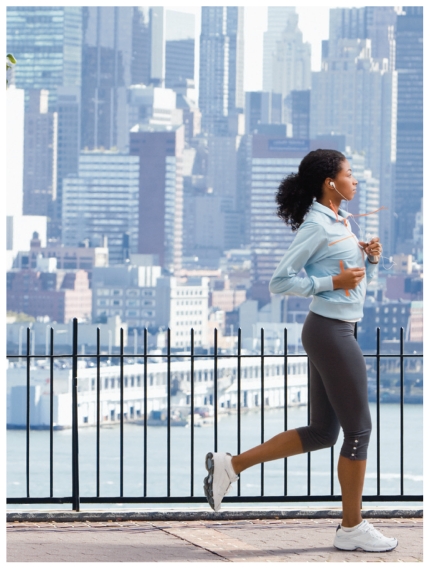
Time of day and location affect air pollution exposure during exercise.

